# Theoretical model for neurovascular coupling via interactions of NO, EET, and 20-HETE

**DOI:** 10.1186/1471-2202-14-S1-P180

**Published:** 2013-07-08

**Authors:** Hyuk Kang, Jung-Min Lee, Misun Kim, Dong-Uk Hwang

**Affiliations:** 1 National Institutes for mathematical Sciences, Daejeon, 305-811, Republic of Korea; 2 Department of Electrical Engineering, Korea Advanced Institute of Science and Technology, Daejeon, 305-701, Republic of Korea

## 

All neurons in the brain need the energy sources such as glucose and oxygen for their functions such as recognition, decision, and behavior regulation. Those energy sources are supplied directly from the capillary around the neuron, or are transported indirectly via the astrocyte. Especially, since more activated neuron consumes more energy sources, the neuron elevates intakes of glucose and oxygen via increasing the blood flow of vessels around itself. Therefore, the blood flow in the brain is important medically, and is one of major parameters in measurement of the brain activity such as functional magnetic resonance image (fMRI) and Near-infrared Spectroscopy (NIRS).

In many experiments, it has been discovered that neuron and astrocyte regulates the brain blood flow via the generated vasoactive agents from them [1]. Usually, vasoactive agents are directly generated from the activating neuron, or are produced indirectly from the stimulated astrocyte by the released neurotransmitter from the neuron. Those vasoactive agents dilate or contract the blood vessel via action on its smooth muscle cells, and there are several kinds of them: NO, EET, prostaglandin, 20-HETE, and so on. But vasoactive agents have no additive effect [2]. For example, although NO inhibitor is added to the blood with EET inhibitor, the inhibition effect shows no increase [3]. In addition, NO sometimes inhibits the effects of EET or 20-HETE [1,4]. Therefore, the temporal or spatial roles for functional hyperemia by vasoactive agents, neuron and astrocyte become an object of attention, and, if interactions and equivalence between dilation and contraction materials are known in their system, it provides the information about the detailed neurovascular modulation.

As previously described, although the mathematical model should importantly consider the coupled effect of vasoactive agents, present modeling studies have been represented via independent interaction between vasoactive agent and vessel. Therefore, we propose the multi-compartment mathematical model, which consists of postsynaptic neuron, astrocyte, and smooth muscle cell of vessel. It is described by the following sequence: 1) the activated presynaptic neuron triggers glutamate release, 2) the glutamate binds to the metabotropic receptor of astrocyte or NMDA receptor of postsynaptic neuron, 3) the bounded glutamate increases the calcium concentration in both astrocyte and neuron, 4) the calcium concentration increase creates arachidonic acid in astrocyte and NO in neuron, 5) the arachidonic acid is metabolized to transform EET or 20-HETE, which are inhibited partly by NO, 6) EET increase the K+ channel conductance of smooth muscle cell (SMC), 7) 20-HETE decreases the K+ channel conductance of SMC, 8) SMC is hyperpolarized via the outward K+ influx, 9) voltage-dependent Ca2+ channel of SMC is deactivated, and Ca2+ concentration decreases, 10) the contraction force of SMC decreases, so that vessel dilates. From the mathematical model, we obtain the simulation results for the direct effect of neuronal derived NO, the inhibition of 20-HETE (vasoconstriction agent) by NO, the interaction of EET (astrocyte derived vasodilation agent), and their corresponding vessel response, as an expansion of the EET generation model in Bennet's study [5].
